# Evaluation of Periaortic Adiposity and Metabolic Disorders in Obese Children

**DOI:** 10.4274/jcrpe.2308

**Published:** 2016-03-01

**Authors:** Beray Selver Eklioğlu, Mehmet Emre Atabek, Nesibe Akyürek, Hayrullah Alp

**Affiliations:** 1 Necmettin Erbakan University Faculty of Medicine, Department of Pediatrics, Division of Pediatric Endocrinology and Diabetes, Konya, Turkey; 2 Konya Training and Research Hospital, Clinic of Pediatric Endocrinology and Diabetes, Konya, Turkey; 3 Malatya State Hospital, Clinic of Pediatric Cardiology, Malatya, Turkey

**Keywords:** obesity, periaortic fat thickness, Atherosclerosis, children, adolescents

## Abstract

**Objective:**

To evaluate the relationship between periaortic fat thickness (PAFT) and parameters involved in the development of metabolic complications of the cardiovascular system in obese children and to assess the usefulness of echocardiographic measurements of PAFT in correlation with cardiovascular risk factors.

**Methods:**

The study was conducted with 263 obese and 100 healthy children and adolescents. PAFT was measured with echocardiography method which was recently performed in obese children and adolescents.

**Results:**

PAFT was significantly higher in the obese group (0.258±0.031 mm) than in the control group (0.137±0.032 mm) (p<0.001). In multivariable regression analysis, body mass index-standard deviation score and total body fat were predictors of PAFT. The area under the receiver operating characteristic curve was 0.989 and was quite significant at p<0.001. PAFT above 0.179 mm was determined as the cut-off value in obese children and adolescents (sensitivity=1, specificity=0.97).

**Conclusion:**

The measurement of PAFT in obese children and adolescents may be a good method to reveal the presence of early cardiovascular risk.

**WHAT IS ALREADY KNOWN ON THIS TOPIC?**

Periaortic adiposity is a strong and new risk factor for cardiovascular disease. Studies were carried out in adult groups using multidetector computed tomography or magnetic resonance imaging.

**WHAT THIS STUDY ADDS?**

Applying echocardiography in childhood for measuring periaortic fat thickness. Determining the presence of early cardiovascular risk in childhood with a non-invasive technique beside classic methods.

## INTRODUCTION

Obese children are candidates for accelerated development of vascular disease due to obesity-induced risk factors. Atherosclerosis, an inflammatory condition, lies at the foundation of cardiovascular conditions. Inflammation also develops on the vessel wall, similar to fatty tissue (1,2).

Atherosclerosis can be identified in its early stage by ultrasonographic or echocardiographic measurement of the intima media thickness of the carotid artery or of other large arteries. The thickness of the carotid intima media is increased in obese children, however, there are conflicting data and findings in the literature with regard to the factors behind this increase (3).

Although subcutaneous fatty tissue contains the majority of body fat, visceral adiposity due to obesity plays an important role in the development of the metabolic syndrome and in the pathogenesis of atherosclerosis. Periadventitial fat accumulation is the localized form of body fat in large blood vessels. Periaortic adiposity is a subtype of perivascular adiposity and is an important indicator of atherosclerosis, which is a critical complication of obesity (2,4,5,6). In adult studies using multidetector computed tomography or magnetic resonance imaging (MRI), periadventitial fat accumulation was considered to be strong and a new risk factor for cardiovascular disease.

The main purpose of this study was to measure periaortic fat thickness (PAFT) by echocardiography and to reveal the relationships between PAFT and metabolic data. MRI examination is expensive and not easily available in most clinical settings. Echocardiography is a noninvasive method which can be used to image aortic fat without radiation exposure. We believe our study will be helpful in the evaluation of endocrinological and cardiovascular complications in the monitoring of obese children.

## METHODS

The study population consisted of 263 obese children and adolescents (129 females, 134 males, aged 11.42±2.69) who presented to the Pediatric Endocrinology Outpatient Clinic of the Faculty of Medicine at Necmettin Erbakan University in Konya, Turkey. Obesity was defined as a body mass index (BMI) greater than the 95^th^ percentile for age and gender (7). Exclusion criteria were the presence of chronic diseases, having genetic or endocrinological diseases, having heart disease, or use of any medication. The control group in this study consisted of 100 children and adolescents (45 females and 55 males, aged 12±2.51 years). Healthy children and adolescents with normal percentiles of weight and height were selected as control group.

The study was approved by the local ethics committee (2010/034) and designed prospectively. The study was conducted in accordance with the guidelines proposed in the declaration of Helsinki.

All participants underwent a thorough physical examination by the same pediatric endocrinologist. Tanner stage based on breast stage and pubic hair development in girls and on genitalia development in boys was assessed in each child.

Height was measured to the nearest 0.5 cm on a standard height board, and weight was determined to the nearest 0.1 kg on a standard physician’s beam scale with the subject dressed only in light underwear and no shoes. BMI was calculated as weight (in kilograms) divided by height (in meters) squared. Waist circumference (WC) was measured at the level of the umbilicus with the patient standing and breathing normally. WC was evaluated using the percentile curves for WC of healthy Turkish children (8). The hip circumference (HC) was estimated on the basis of the widest diameter passing through the most protruding point of the gluteus maximus and over the symphysis pubis. Waist/hip (WC/HC) ratio was determined by dividing WC to HC. Blood pressure was measured with a standard mercury sphygmomanometer after the subjects had rested for at least 10 minutes. Blood pressure threshold values were evaluated with reference to the normal values reported for children in the National High Blood Pressure Education Program Working Group in 2004. Casual systolic blood pressure (SBP) and diastolic blood pressure (DBP) values more than 95^th^ percentile for age, sex, and height were defined as hypertension (9).

Serum fasting glucose, fasting plasma insulin, total cholesterol, triglyceride, low-density lipoprotein (LDL), and high-density lipoprotein (HDL) cholesterol levels were estimated.

The homeostasis model assessment of insulin resistance (HOMA-IR; fasting insulinxfasting glucose/22.5) was used as an index of insulin resistance (10). Insulin resistance is defined as a HOMA-IR of greater than 2.5 in the prepubertal group and 3.16 in the pubertal group (11,12).

Total body fat was measured by bioelectricimpedance analysis (Model MC 180, MA Multi-Frequency Body Composition Analyzer; Tanita, London, UK) with correction for light indoor clothing.

**Figure 1:**
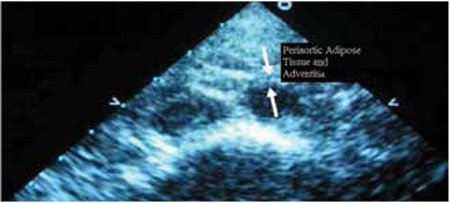
Image of periaortic adipose tissue and adventitia on echocardiography

Echocardiographic examinations were performed with a Philips Hewlett-Packard Sonos 5500, using 12 MHz flat probes, according to the American Association of Echocardiography Pediatric and Congenital Heart Disease Council’s Standard imaging techniques (13). Periaortic adipose tissue was measured from the periaortic tissue to the muscular layer of the abdominal aorta which represents periaortic tissue with adventitial layer of the abdominal aorta. Measurement of periaortic adipose tissue should be taken with adventitia (Figure 1) because in deep tissue it couldn’t be differentiated exactly with echocardiography and ultrasonography.

PAFT was measured in sagittal and axial planes at L1-2 level, proximal to the iliac bifurcation in the supine position.

Evaluations were made three times by pediatric cardiologists. The mean PAFT values were recorded. Reliability tests were also performed.

### Statistical Analysis

Normality was checked. Data are expressed as means ± standard deviation. Student’s t-test and chi-square test were used.

Multiple regression analysis was performed.

Reliability testing was done to evaluate PAFT measurements in the obese and control groups. Compliance (reliability) within observers and between observers was assessed for axial and sagittal measurements. Measurement of PAFT were coherent for both intra class and inter class evaluation determined by intraclass correlation coefficient with 95% confidence intervals.

## RESULTS

Reliability of axial and sagittal measurements of PAFT are shown in Table 1 for inter-observer and in Table 2 for intra-observer differences.

Interpretation of calculated levels of compliance by intraclass correlation coefficients:

0-40: measurements compatible (consistent),

41-60: measurements of harmony (consistency) low,

61-80: measurements sufficiently compatible (consistent),

81-100: measurements quite consistent.

The mean age of the subjects in the obese group was 11.42±2.69 years and that of the control group was 12±2.51 years. 65% of the obese group and 74% of the control group were pubertal. Demographic and anthropometric parameters of obese andcontrol groups are shown in Table 3.

**Figure 2:**
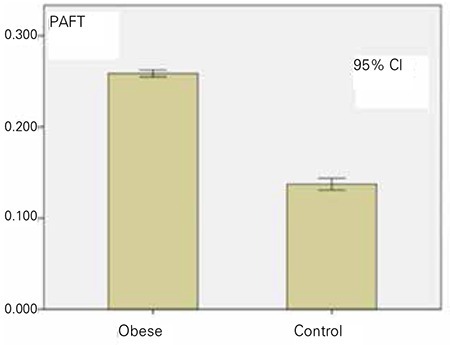
Periaortic fat thickness of obese and control groups. PAFT: periaortic fat thickness, CI: confidence interval

PAFT was 0.258±0.031 mm in the obese group and 0.137±0.032 mm in the control group and this was statistically significant difference (p<0.001) (Figure 2). PAFT was not statistically different according to sex or pubertal status. Cardiovascular and laboratory parameters of obese and control groups are shown in Table 4.

In the obese group, 99 cases (37.6%) had SBP elevation. In the control group, there was no blood pressure elevation. SBP and DBP values in the obese group were statistically significantly higher than in the control group. Dyslipidemia was detected in 46.1% of the patients. Between the groups with and without dyslipidemia, PAFT was not statistically significantly different (p=0.95). In 41.7% of obese patients, insulin resistance was detected. PAFT in patients with insulin resistance was not significantly higher than in the group without insulin resistance (p=0.44).

The significant correlations between PAFT and clinical and laboratory parameters are shown in Table 5.

In multivariate regression analysis, the only predictors of PAFT were BMI-SDS (β:0.47, p<0.001) and total fat percentage (β:0.37, p<0.001).

The area under the receiver operating characteristic curve was 0.989 and was quite significant at p<0.001. PAFT above 0.179 mm was determined as the cut-off value for obese children and adolescents (sensitivity=1, specificity=0.97).

## DISCUSSION

The most important cardiovascular problem observed in obesity is early development of atherosclerosis. Facilitators of development of atherosclerosis are type 2 diabetes and the presence of hypertension and dyslipidemia. An increase in visceral adipose tissue disrupts metabolic balance, enhances generation of proinflammatory and prothrombotic substances, and increases the risk of atherosclerosis (14).

Coronary atherosclerosis is the most well-known pathology. This process begins in childhood and can be irreversible at this stage. Irreversible fatty lines, rather than atherosclerosis, generally develop in children. Studies indicate that the severity of atherosclerosis in children and young adults is associated with the same risk factors determined in adults. Many studies have shown that fatty lines and fibrous plaques in coronary arteries of adolescents and thickening in vessel intima were determined from the age of 5 years (15,16).

Periaortic adiposity is an important indicator of atherosclerosis that also begins at an early period (17). Another component of abnormal body fat accumulation is accumulation of ectopic fatty tissue. It surrounds the organs and vessel structures. Perivascular adiposity is a type of ectopic adiposity. It is believed to have a local pathogenic effect on blood vessels. Periaortic adiposity is a subtype of perivascular adiposity and only publications using measurement by multi-sectional computer tomography are currently available (4,6). Measurements in these studies are in limited numbers and are experimental.

In this research PAFT values were taken with echocardiography method. PAFT was found to be 0.258±0.031 mm in the obese group and 0.137±0.032 mm in the control group (p<0.001). Obese group had significantly higher PAFT values. The threshold value for PAFT was determined to be 0.179 mm in obesity.

Lehman et al (18) reported in their study that thoracic adiposity was related with metabolic risk factors. The relationship between visceral adipose tissue and periaortic adiposity is not known. In our study, supporting the findings of Lehman et al (18) a positive correlation was found between PAFT and cardiovascular risk factors, namely, SBP and DBP, total cholesterol, LDL cholesterol, and triglyceride. Ruberg et al (19) found PAFT to be higher in an obese group than a control group and to have a positive correlation with BMI and a negative correlation with HDL cholesterol in their study performed with MRI. Schlett et al (20) found PAFT to positively correlate with BMI and WC.

Britton et al (4) used computed tomography in adult studies and showed the relation of thoracic aortic fat, cardiac and metabolic disorders.

In cases who had increased aortic adiposity, BMI, WC and visceral adiposity were markedly higher. In this present study, we also found a positive correlation between PAFT and WC, an important indicator of visceral adiposity. All our data support the aforementioned studies.

Thanassoulis et al (5) reported that increased periaortic adiposity is related to aortic remodeling. They emphasized that local adiposity in the aorta caused aortic remodeling to a greater extent than the systemic effects of obesity. We found that among the risk factors only BMI-SDS and total fat had effects on PAFT, which is consistent with this hypothesis.

The fact that periaortic adiposity is correlated with all cardiovascular risk factors in our study indicates that it only defines the cases who are metabolically obese and who carry cardiovascular risks. As shown in our study, a lack of differences based on gender or pubertal stage indicates that periaortic adiposity may possibly be used as a standard method.

The mechanisms responsible for the development of local adiposity in the vessels are not currently clear and the role of this local adiposity in the development of insulin resistance and metabolic syndrome is still being reviewed. In support of thisrelationship, PAFT was found to have a positive correlation with serum insulin level and HOMA-IR in our study.

Insulin resistance provides groundwork for the development of atherogenic dyslipidemia, prothrombotic and proinflammatory conditions. It is reported that insulin resistance observed in obesity also contributes to the development of hypertension (21). Coronary artery disease is significantly associated with fasting insulin levels. Other findings in our study, such as differences between the obese and control groupsregarding insulin level and HOMA-IR, findings independent of blood glucose level, also support the importance of insulin resistance in the development of these pathologies.

In obesity, sympathetic nervous system activation occurs and catecholamine secretion increases as a result of excessive intake of calories through foods rich in fat and carbohydrates. Blood pressure elevates with increased catecholamines (22). In our study,elevated SBP was detected in 37.6% of obese subjects andwas statistically significantly higher than in the control group.

Reinehr et al (23) reported the frequency of hypertension in obese children as 38%, and Maggio et al (24) as 47%. In other studies, conducted by the monitoring of blood pressure for 24 hours, this rate was reported to be between 47-60% (24).

Saha et al (25) reported the frequency of insulin resistance as 63%in obese subjects. Viner et al (26) reported a frequency of 30% for dyslipidemia. In our study, we found 46.1% of patients to have dyslipidemia in the obese group. LDL cholesterol, total cholesterol, and triglyceride levels in obese cases were higher and HDL cholesterol level were lower than in the control group. This suggests that dyslipidemia is important in the development of obesity.

Various studies indicate that BMI and WC are important indicators of obesity and body fat distribution and that WC is an important indicator of cardiovascular risk (27). That WC is correlated with PAFT in our study also supports these findings.

The relationship between local fat distribution and cardiometabolic complications is not known. Increased WC-HC ratio is believed to represent an increase in abdominal adipose tissue. Compared to other anthropometric measurements, it is reported to have a positive correlation with cardiovascular disease. Many studies support this relation (21). In our study, WC/HC ratio was found to be increased in obese cases and a positive correlation was determined with PAFT. These findings support the reports in the literature.

Our results also indicate that evaluation of PAFT is important for early diagnosis of atherosclerosis in obese cases, the most important factors for PAFT, independently from additional metabolic risks, being BMI and total fat mass.

The limitation of our study was that the findings were not validated with standard techniques such as MRI.

This study showed the usefulness of echocardiographic measurement of PAFT in correlation with cardiovascular risk factors. Echocardiography allows good delineation of normal abdominal aortic anatomy including the recognition of vessel layers, especially when they are thick and contain fat. We also showed that the measurement of PAFT had good intra-operator and inter-operator reliability. PAFT measurement with conventional echocardiography in obese cases may be useful for assessing cardiovascular risk at earlier ages.

## Ethics

Ethics Committee Approval: Necmettin Erbakan University Ethics Committee (Approval number: 2010/034), Informed Consent: Verbal consent.

Peer-review: External and Internal peer-reviewed.

## Figures and Tables

**Table 1:**
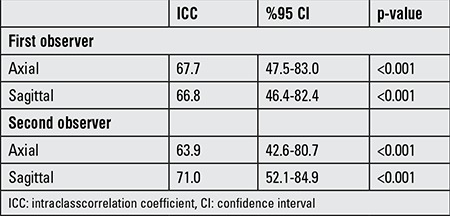
Intra-observer reliability table

**Table 2:**
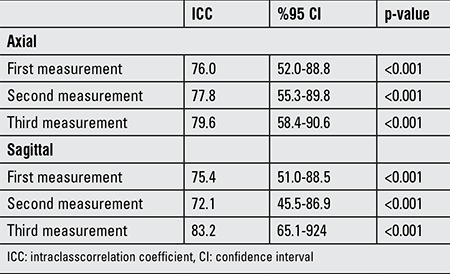
Inter-observer reliability table

**Table 3:**
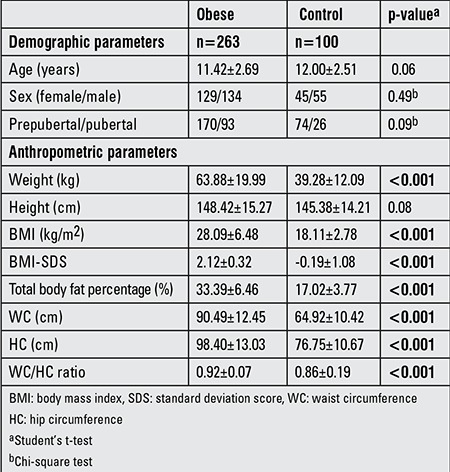
Demographic and anthropometric parameters of obese and control groups

**Table 4:**
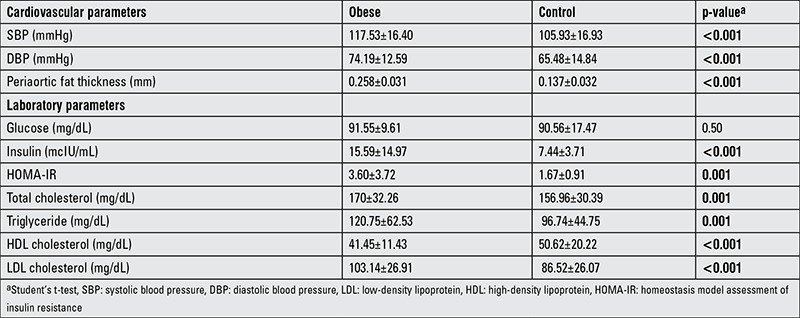
Cardiovascular and laboratory parameters in the obese and control groups

**Table 5:**
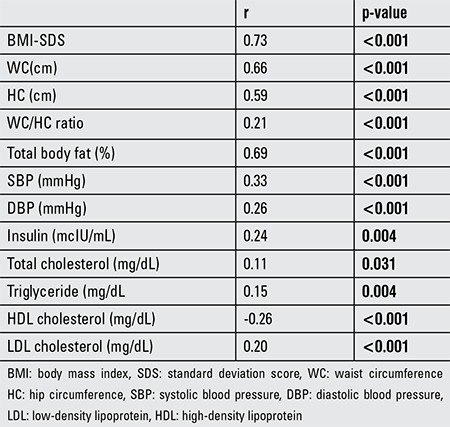
Correlations between periaortic fat thickness and clinical/laboratory parameters
